# The Desert in Bottles: The Culturable Microbiome of the Atacama Desert's Grit Crust

**DOI:** 10.1111/1758-2229.70194

**Published:** 2025-09-19

**Authors:** Patrick Jung, Laura Briegel‐Williams, Lina Werner, Emily Jost, Rebekah Brand, Karen Baumann, Michael Lakatos

**Affiliations:** ^1^ XCEL – Extreme Cryptogam Ecology Lab University of Applied Sciences Kaiserslautern Kaiserslautern Germany; ^2^ Department of Integrative Biotechnology University of Applied Sciences Kaiserslautern Pirmasens Germany; ^3^ Geo Laboratory University of Vechta Vechta Germany

**Keywords:** biocrust, cultivation, cyanobacteria, desert, green algae, non‐lichenised fungi

## Abstract

Biological soil crusts (biocrusts) are associations of microorganisms coexisting in the top millimetres of soil, which are found predominantly in arid biomes. Recently, a new type of biocrust, termed grit crust, of the coastal range of the Atacama Desert was discovered. Here, we explore the culturable microbiome of the grit crust based on an integrative isolation approach combining morphological and phylogenetic analyses, focusing on cyanobacteria, green algae and the often overlooked non‐lichenised fungi with climate records and soil data. The 122 generated isolates from four contrasting locations were distributed over three organismic groups: cyanobacteria (38), green algae (26) and non‐lichenised fungi (58). The distribution of the organisms among the four locations followed a water availability gradient as shown by relative air humidity data, resulting in communities shaping the biochemistry of the substrate in terms of texture, carbon and nitrogen contents. Novel species, genera and the functional roles of the different organisms within the biocrust environment are discussed. The high abundance of endemic and presumably new species, including five potentially new genera within the cyanobacterial order of the Chroococcidiopsidales—not found in other deserts—underpins the uniqueness and further distinguishes the grit crust from other biocrusts in extreme environments.

## Graphical abstract

The grit crust represents a unique biocrust type of the coastal Atacama Desert. Cyanobacteria, green algae and free‐living fungi were isolated along a fog gradient. Phylogenetic relationships indicated novel taxa and framed an indigenous microbiome. Microclimate and soil data support functional groups related to sites.

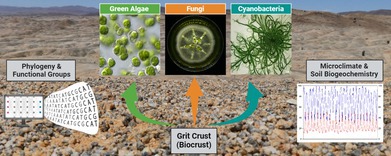

## 1 Introduction

Desert ecosystems, characterised by their limited water availability and challenging climatic conditions, host a unique and often overlooked ecological component of vegetation known as biological soil crusts (biocrusts) (Reed et al. 2019; Sosa‐Quintero et al. 2022). These intricate communities of microorganisms, predominantly comprised of (cyano)bacteria, algae, lichens, mosses and fungi, colonise the uppermost layer of soil surfaces in arid and semi‐arid regions worldwide, covering at least 12% of the Earth's surface and about 30% of all dryland soils (Rodriguez‐Caballero et al. 2018). The collective activities of these diverse (micro)organisms contribute to soil stabilisation through protection against erosion by wind and water (Eldridge and Leys 2003; Belnap et al. 2011; Faist et al. 2017), to nutrient cycling as ecosystem engineers, and as primary producers fixing carbon and nitrogen (Xiao et al. 2022; Barrera et al. 2022).

Biocrusts composed of poikilohydric organisms serve as pioneers in harsh desert landscapes, where they have evolved mechanisms to thrive under water‐limited conditions, surviving where most vascular plants cannot (Meier et al. 2021; Mackelprang et al. 2022). Besides their extremotolerant to extremophile environments (Jung et al. 2022), biocrusts encompass structural and diverse complexity (Chilton et al. 2018), as well as cross‐phylum symbiotic interactions (Nelson et al. 2021), and reflect the adaptation of constituent organisms to the challenging ecological constraints of deserts.

Regarding the microbial diversity of biocrusts, attention has most often focused on a group of commonly occurring cyanobacteria, green algae and lichens, including their ecological functions within the consortia. Whether Atacama grit crust follows comparable patterns of diversity and function is yet to be realised. One of the most frequent cyanobacterial biocrust‐forming species, for example, is *Microcoleus vaginatus* , which plays significant roles during the concatenation of soil particles and the formation of crusts due to the secretion of extracellular polymeric substances (EPS) and its rope‐like development of filaments (Xiao et al. 2023). In addition, heterocytous cyanobacteria of the Nostocales, such as *Nostoc* spp. or *Scytonema* spp., have also often been identified in biocrusts where their nitrogen‐fixing ability significantly contributes to the nitrogen budget (Williams et al. 2018). Besides filamentous cyanobacteria, unicellular taxa such as *Chroococcidiopsis* have also been identified in hot and cold deserts (Bahl et al. 2011), often associated with biocrusts (Lan et al. 2021). For this reason, for decades, *Chroococcidiopsis* strains have often been considered extremophiles (Billi et al. 2010; Aguiló‐Nicolau et al. 2023), but their extremophile habitat niche has recently been challenged by molecular studies indicating a misconception of this genus due to its uncertain taxonomic status (Antonaru et al. 2023). Among microalgae, free‐living unicellular algae such as *Stichococcus*, *Chlorella* and *Bracteacoccus* can also be constituents of biocrusts, where they contribute to primary productivity (Samolov et al. 2020), while other members, such as *Klebsormidium*, stabilise the soil due to their filamentous growth form and mucilage production (Mikhailyuk et al. 2015; Belnap and Büdel 2016).

These groups of microalgae are the main primary producers in biocrusts, and their communities are also associated with free‐living fungi, which often have phytopathogenic lifestyles such as members of the genera *Cladosporium* or *Alternaria* (Bates and Garcia‐Pichel 2009; Abed et al. 2013). The hyphal networks of those fungi not only add stability to the crust, but they also decompose carbon compounds, perform nitrification and denitrification and store nutrients intracellularly (Hawkes 2003; Rudgers et al. 2018).

The highest degree of microbial complexity can be provided by lichens, a symbiotic organisation of green algae and/or cyanobacteria with fungi, which are frequent residents of biocrust habitats (Rosentreter et al. 2016). Common species include *Psora decipiens*, *Enchylium tenax* and *Diploschistes diacapsis* (Williams et al. 2017; Green et al. 2018). Biocrust lichens are often associated with the photobiont *Trebouxia*, a green algal genus which is one of the most frequently observed green algal photobionts in general (Muggia, Candotto‐Carniel, et al. 2017; Muggia, Kopun, et al. 2017; Moya et al. 2020). Lichens have now been recognised as self‐sustaining micro‐ecosystems because they harbour a vast diversity of additional microorganisms, some of which play significant roles in the symbioses (Hawksworth and Grube 2020). Such lichens contribute to carbon fixation and nutrient cycling, further influencing the availability of essential elements for other organisms in the biocrust ecosystem (Maestre et al. 2013; Sancho et al. 2016; Tian et al. 2023). The role and significance of lichens and their photobionts within the biocrust community depend on the biocrust type and biome, as in most reported cases, such lichens grow in patches (Concostrina‐Zubiri et al. 2018). Recently, lichens of the family Caliciaceae, including their trebouxioid photobionts (resembling or related to *Trebouxia*), were identified as the main organismic constituent forming a unique type of transitional stage between a saxicolous community (rock‐dwelling or growing on rocks) and a biological soil crust, the biocrust type—‘grit crust’ (Jung, Brand, et al. 2024). The grit crust is the first ecosystem found to be dominated by symbiotic algae, which is responsible for a significant biomass build up, bio‐weathering activity (Jung, Baumann, Emrich, et al. 2020), ecophysiological performance and carbon fixation in the coastal Atacama Desert (Jung, Baumann, Lehnert, et al. 2020). In the grit crust, lichens colonise granitoid pebbles of 2–6 mm diameter and reach such a high density that their colours and structures form blackish spots of a checkerboard pattern visible to the naked eye across the landscape (Jung, Baumann, Lehnert, et al. 2020). Although lichens and lichenised green algae of the genus *Trebouxia* were found to make up the majority of the grit crust community, initial metabarcoding revealed other groups of associated, free‐living microorganisms that had not previously been studied (Jung, Brand, et al. 2024).

On the one hand, our research can be divided into broader aspects of biodiversity such as (i) what is the taxonomic and morphological diversity of cyanobacteria, green algae and non‐lichenised fungi in the grit crust of the Atacama Desert?, (ii) how does the microbial composition of the grit crust compare to that of other biocrusts in arid or extreme environments?, (iii) what novel taxa (species or genera) can be identified in the grit crust, and how do they phylogenetically relate to known organisms? On the other hand, we formulated specific questions such as (i) what ecological or functional roles do non‐lichenised fungi play in these newly described biocrust systems?, (ii) is *Microcoleus* spp. a dominant cyanobacterial constituent of the grit crust similar to other biocrusts?, and (iii) can other members of potential green algal photobionts be isolated, such as *Myrmecia* spp. or *Asterochloris* spp.?

Further exploration of this diversity is of major importance for the research community since the grit crust, as a unique type of biocrust, adds a new level to our general understanding of biocrusts and the functions of microorganisms in extreme habitats. This has also recently been highlighted during a refined, contemporary definition of the term biological soil crust (Weber et al. 2022).

In contrast to some molecular techniques, such as standard metabarcoding, which may recover DNA from both living and dead organisms, classical culture‐dependent approaches ensure the isolation of metabolically active and viable microbes. This allows for the functional and physiological characterisation of taxa that are demonstrably alive and potentially ecologically active within the soil system (Carini et al. 2016; Rippin et al. 2018; Francioli et al. 2021). While advanced molecular tools such as stable isotope probing or metatranscriptomics can target active populations, they require additional resources and experimental setups. Our approach provides a practical and targeted strategy to investigate viable organisms, especially in an environment where novel taxa are expected and functional characterisation is a key objective. Usually, only a small fraction of the total microbial diversity can be cultivated (Epstein 2013), but for those organisms that are successfully isolated, the combination of molecular phylogeny and classical morphological comparisons enables high‐resolution identification, often down to the species level (Jung et al. 2019; Samolov et al. 2020; Jusko and Johansen 2023). This also includes the identification and description of novel species with significant functional roles within biocrusts (Skoupý et al. 2022; Bethany et al. 2022) or a characterisation of such strains for certain biotechnological applications (Karimi et al. 2023; Miralles et al. 2023). Over the last few decades, culture‐dependent investigations have resulted in the organismic description of different biocrusts across the globe, but these studies mainly focused on single groups such as cyanobacteria and/or green algae (Cano‐Díaz et al. 2018; Roncero‐Ramos et al. 2019; Mikhailyuk et al. 2021). In general, free‐living fungi of biocrusts have rarely been investigated or isolated (Bates and Garcia‐Pichel 2009; Abed et al. 2013) and have so far never been investigated in a polyphasic study together with other biocrust organisms such as algae. Despite aspects of microbiological biocrust diversity, jointly isolating microorganisms across several phyla from such complex systems is pivotal for subsequent investigations, paving the way for a deeper understanding of possible interactions (Nelson et al. 2021), nutrient exchange (Aanderud et al. 2018), adaptation strategies (Giraldo‐Silva et al. 2020) or population dynamics (Kedem et al. 2021).

This work will complement the knowledge on the biodiversity of microorganisms contributing to the grit crust, which until now has focused on lichens and their photobionts (Jung, Brand, et al. 2024). Unialgal and axenic fungal isolates were first established, followed by characterisation based on morphological features as well as their phylogenetic positions. In addition, the diversity of the grit crust is compared to other biocrust environments and the functional role of the isolated non‐lichenised cyanobacteria, green algae and fungi within this biocrust type is discussed.

## 2 Materials and Methods

### 2.1 Sampling Location

The National Park Pan de Azúcar, situated within the arid expanse of the Atacama Desert in northern Chile, spans approximately 440 km^2^ and encompasses a diverse range of ecological landscapes. The National Park is a crucial site for scientific inquiry and preservation efforts in the context of arid and coastal ecosystems, which was highlighted during the research project EarthShape. As a result, geological, ecological and climatological information on the National Park can now be accessed (Baumann et al. 2018; Bernhard et al. 2018; Samolov et al. 2020).

### 2.2 Sampling

**FIGURE 1:**
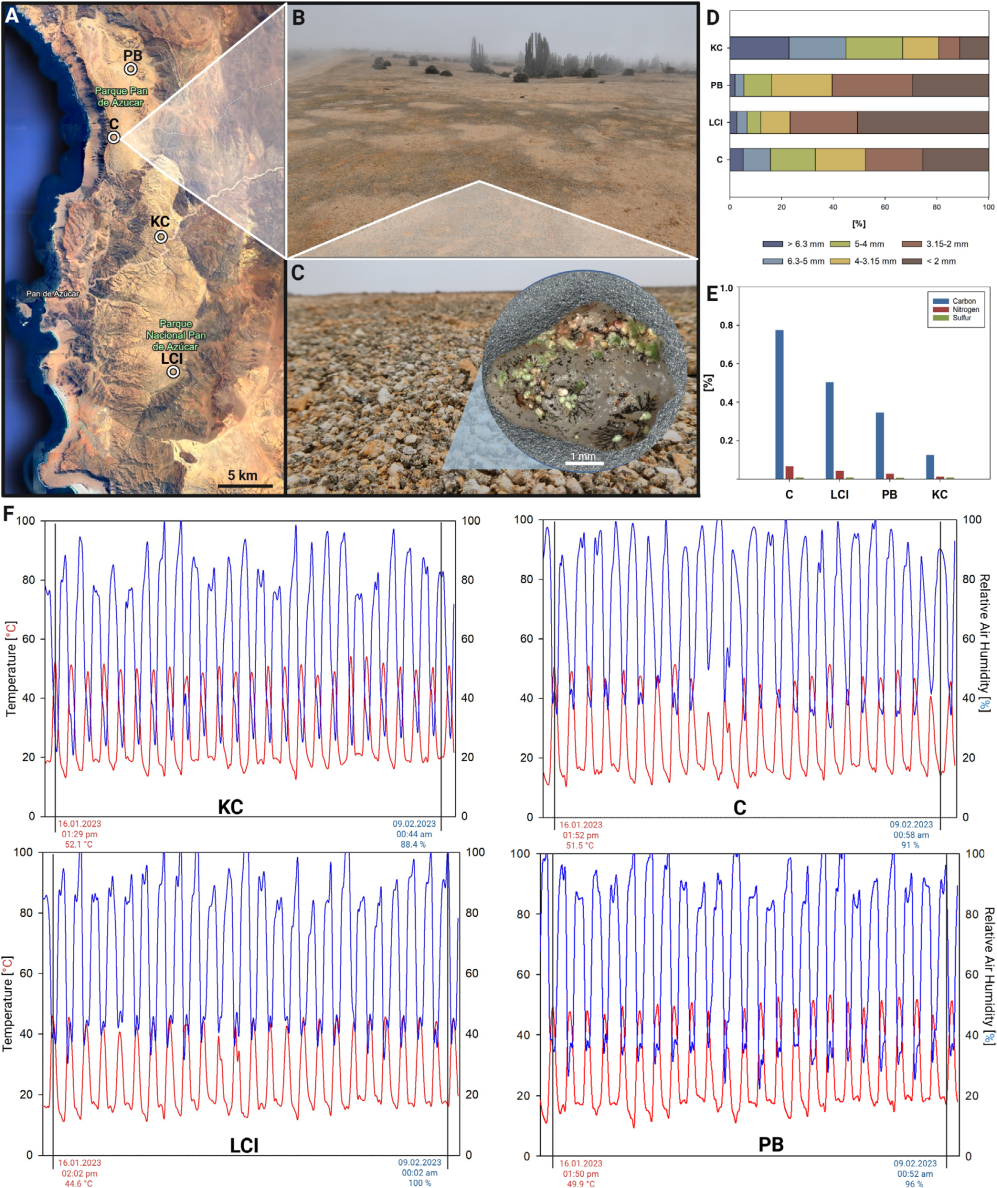
Photographs, soil‐ and microclimate data of the four sampling sites and Venn diagrams of isolated taxa. (A) Map showing the four sampling sites C (humid), KC (dry), PB and LCI (intermediate) in the National Park Pan de Azúcar. (B) Photograph showing blackish‐whitish pattern caused by the grit crust at location C. (C) Detailed photograph of a blackish spot showing the grit crust with an insert showing a single, hydrated grit stone colonised by greenish lichens. (D) Texture of biocrust substrate from the four sites. (E) Carbon, nitrogen and sulphur content of biocrusts from the four sites. (F) Microclimate data of each site measured on ground level showing temperature and relative air humidity over 25 days (mid‐January to mid‐February 2023). In each graph, the daytime of the highest temperature during the first day and the highest relative air humidity during the last day are marked.

Within the National Park Pan de Azúcar, 11 sampling sites were chosen during related research projects where permanent 1 m^2^ plots had been established, including one iButton Hygrochron temperature/humidity logger (DS1923, Maxim Integrated, CA, USA) monitoring relative air humidity and temperature at the top first cm level every 30 min, representing the micro‐climate at the interface between soil and air (Jung, Brand, et al. 2024). Four of the 11 sampling sites had been selected for this study because they marked significant locations (Figure 1a): site KC (26°06′30″; 70°33′01″) was devoid of vegetation and marked the driest area within the National Park located 12 km off the coast with almost no fog influence (referred to as ‘dry’ site). Site C (26°00′26″; 70°36′21″) was situated only 100 m off the coastal ridge, where fog was frequently present and resulted in a higher level of vegetation made up of cacti and *Euphorbia* bushes (referred to as ‘humid’ site). Both sites represented the extremes in terms of microbial density since KC was weakly colonised and C had the densest grit crust appearance. The sites PB (26°12′09″; 70°33′31″) and LCI (25°57′02″; 70°36′24″) represented intermediate locations with a flat landscape without vascular vegetation, partly influenced by fog and with a medium coverage of the grit crust. At all four sampling sites, three soil samples of 50 g top‐soil layer (1 cm) each were collected with a spatula during February 2021 (summer season) for the isolation approach and the determination of soil texture in the close vicinity of the 1 m^2^ plots and stored in plastic containers. In addition, one sample from the first top centimetre of a 10 cm^2^ subplot within each plot was taken for the soil biochemistry analysis. All samples were air‐dried and stored in the dark at room temperature until further analysis.

### 2.3 Texture and Soil Biochemistry

Soil texture was determined on a combined sample of three field replicates from the first centimetre using sieving procedures after Blume et al. (2010).

For elemental analyses, samples were ground to < 0.5 mm using a mixer mill (MM 200, Retsch, Haan, Germany) operated at 30 Hz for 5 min with steel beads. Each sample was analysed in duplicate. Total C, N and S content was obtained by dry combustion using an elemental analyzer (VARIO EL, Elementar Analysensysteme GmbH, Hanau, Germany).

### 2.4 Cultivation of Green Algae, Cyanobacteria and Non‐Lichenised Fungi

To identify biocrust‐forming photosynthetic and non‐photosynthetic organisms, an integrative approach was applied. The cultures for the three organism groups, cyanobacteria, green algae and non‐lichenised fungi, were started from the same samples. About 5 g of biocrust material was filled in three sterile plastic tubes per location. These were filled with 10 mL sterile Ringer's solution (Reddy et al.) and mixed for 10 min on a vortex instrument. Afterwards, the tubes were incubated at room temperature for 30 min and again mixed on a vortex instrument for 10 min before a dilution series of 1:1, 1:10 and 1:100 was prepared. From each dilution sample, 100 μL were streaked out on three 6 cm agar plates filled with (i) 0.9% solidified BG11 (Stanier et al. 1971) to promote the growth of cyanobacteria, (ii) 0.9% solidified BBM (Bischoff and Bold 1963) for green algae and (iii) 1% Sabouraud agar for fungi (2% glucose and chloramphenicol; Sifin Diagnostics, Berlin, Germany).

All initial cultures were incubated at 25°C in a culture cabinet at 30 μmol photons m^−2^ s^−1^ (CLF Plantclimatics GmbH, Wertingen, Germany) and a 16:8 h light–dark cycle. After 4 weeks, single colonies of algae or cyanobacteria were observed, which were then transferred to new BBM or BG11 agar plates to establish pure clonal cultures. Green algae of the genus *Trebouxia* were ignored during this process because they were found to be predominantly the photobionts of the lichens and the focus of another study (Jung, Brand, et al. 2024). Plates with non‐lichenised fungi were inspected 24 h after incubation, and then daily until no new colonies were observed, and single colonies were transferred to fresh Sabouraud plates. Transfer was repeated until unialgal isolates or axenic fungal and bacterial isolates were generated. The final algal isolates were established as living strains in the research culture collection of the University of Applied Sciences Kaiserslautern (Pirmasens, Germany), stored in culture bottles filled with 200 mL of the corresponding liquid medium. Fungal isolates were cultivated on sterile filter papers placed on solidified Sabouraud medium, which were removed after 1 week of growth, dried at room temperature in sterile plastic boxes and stored in the dark at room temperature.

### 2.5 Enzyme Assay for Non‐Lichenised Fungi

An enzyme assay for the isolated non‐lichenised fungi was conducted in order to understand their lifestyle traits and interpret their functional role within the biocrust community, as demonstrated by Martinez et al. (2016).

The enzymatic activity was measured using the semiquantitative API ZYM test (bioMérieux, France) for 19 constitutively expressed lipid, protein and carbohydrate‐degrading enzymes. The test was applied following the manufacturer's instructions with a suspension of each isolated fungus. Finally, a value of 0–5 was assigned to an enzyme activity according to the instructions corresponding to the established colours, where 0–2 colour intensity corresponds to a negative reaction and 3–5 to a positive reaction.

### 2.6 Morphological and Functional Characterisation

Morphological details were captured by means of various microscopic techniques in order to allow a morphological comparison between the isolated organisms and those described from other biocrusts and literature.

A digital 3D 4K stereo microscope (VHX‐7000, Keyence Deutschland GmbH, Neu‐Isenburg, Germany) was used to capture images during the growth of the algal isolates growing on agar plates after 6 weeks of growth.

Images of isolated fungi were taken 5 days after transfer to new agar plates with a digital camera (TZ 91, Leica, Wetzlar, Germany).

In addition, light and DIC microscopy were used to take detailed images of the isolated algae using a BX51 microscope (Olympus, Tokyo) coupled with a camera (MicroLive, Bremen, Germany) and MicroLive 5 software (MicroLive, Bremen, Germany).

General ecological functions of cyanobacteria and green algae were assigned based on the most updated literature for the corresponding genera and species, while for fungi the FungalTraits database was consolidated (Põlme et al. 2020). This helped to interpret the ecological function of the isolated organisms among the biocrust consortium.

### 2.7 Molecular Characterisation

DNA from the isolates was extracted using the direct PCR protocol as described in Jung, Briegel‐Williams, et al. (2024). Small amounts of biomass (equivalent to three sand grains) were picked from agar plates under a binocular stereoscope using sterile syringes and transferred to PCR‐plastic tubes filled with the lysis buffer. During PCR amplification, the 16S rRNA gene region of cyanobacteria was targeted using the primers B2 and B6 (Boyer et al. 2002). For green algae, the primers EAF3 (Marin et al. 2003) and G800 (Darienko et al. 2019) were used to amplify the SSU gene region, and the primers ITS1f (Gardes and Bruns 1993) and LR3 (Friedl and Rokitta 1997) covered the partial 18S rRNA, ITS1, 5.2S, ITS2 and partial 28S gene regions. Details about primers and cycles are given in Jung, Briegel‐Williams, et al. (2024). The latter primer pair was also used to generate the same gene region of non‐lichenised fungi. All PCRs were conducted following the co‐cycling conditions described by Jung, Briegel‐Williams, et al. (2024), and successful amplification was checked by means of gel electrophoresis. Subsequently, PCR products were cleaned using the NucleoSpin Gel and PCR Clean‐up Kit (Marchery Nagel, New England, Canada) and sent to Genewiz (Göttingen, Germany) for Sanger sequencing.

Sequences of the isolates were compared to those from reference strains in NCBI using BLASTn queries to find the closest relatives. When possible, sequences of authentic strains or strains from public culture collections were used, on which the formal taxonomic description of a specific species was based. Multiple alignments of nucleotide sequences were prepared using the software Mega 11 (Tamura et al. 2021), applying the Muscle algorithm. The evolutionary model that was best suited to the database used was selected based on the lowest AIC (Akaike information criterion) value and calculated in MEGA 11 (Tamura et al. 2021). Phylogenetic trees were constructed using the web server NGPhylogeny.fr (Lemoine et al. 2019), applying the evolutionary model GTR + G + I for all alignments with 500 generations, each to calculate maximum‐likelihood (ML) bootstraps. No significant differences were detected between the 18S and SSU phylogenetic trees of the green algae; thus, a concatenated version was prepared. The final alignment of cyanobacteria comprised 218 sequences, 1018 bp in length; the concatenated alignment of green algal sequences included 115 sequences with a length of 3366 bp, and the alignment of non‐lichenised fungi covered 258 sequences with a length of 1544 bp.

In addition, the program MrBayes 3.2.2 (Ronquist and Huelsenbeck 2003) was used equivalently to calculate Bayesian Inference (BI) with 5,000,000 generations. Two of the four runs of the Markov chain Monte Carlo were made simultaneously, with the trees taken every 500 generations. Split frequencies between runs at the end of the calculations were below 0.01. In case the trees selected before the likelihood rate reached saturation, they were subsequently rejected. There was no significant difference shown between the BI and ML trees; thus, single trees were edited in iTOL (Letunic and Bork 2021) and visualised using Biorender.com. Branches where both analyses resulted in > 80% statistical support were marked with an asterisk.

## 3 Results

### 3.1 Soil Biochemistry and Micro‐Climate

The highest carbon content (Figure 1E) was observed at the humid site C (0.78%), followed by LCI (0.5%) and PB (0.35%), with site KC (dry) showing the lowest content (0.13%). The same declining trend could be observed for nitrogen (0.07% at C, 0.04% at LCI, 0.03% at PB and 0.01% at KC). Sulphur content was < 0.01% at all sites.

At sites C, LCI and PB, 25%–50% of the top 1 cm of substrate had particles < 2 mm, while 25% ranged between 3.15 and 2 mm (Figure 1D). At these three sites, less than 5% of the substrate had a diameter > 6.3 mm. In contrast, at site KC, particles < 2 mm made up less than 10% while almost 25% of the top soil layer was made up by the fraction > 6.3 mm.

Microclimate data of all four sites followed a diurnal day–night pattern during which the lowest temperatures around 10°C–13°C were detected between 5 and 6 AM, and the highest temperatures often exceeding 50°C were observed from 1 to 3 PM (Figure 1F). Lowest relative air humidity at ground level, around 20%–40%, was detected during times with the highest temperatures, while the highest relative air humidity occurred between midnight and 1 AM.

The temperature regime and relative air humidity differed between sites. Location C showed the lowest average temperature (25.98°C ± 12.2°C), followed by LCI (26.04°C ± 11.22°C), PB (27.51°C ± 12.71°C) and the warmest plot at KC (29.24°C ± 12.55°C). The average maximum daily temperature was between 45°C and 51°C; the minimum ranged between 14°C and 17°C. Relative air humidity was lowest at KC (60.4% ± 22.91%), followed by PB (66.24% ± 24.97%) and LCI (67.5% ± 22.53%), while site C, which was closest to the coast, showed the highest average relative air humidity (72.56% ± 25.71%). Highest average daily relative air humidities varied between 98% and 100%, and the minimum was between 29% and 36% at the air‐ground boundary.

### 3.2 Diversity

In total, 122 isolates from four contrasting locations within the National Park Pan de Azúcar were captured, comprising 38 cyanobacterial, 26 green algal and 58 non‐lichenised fungal isolates as summarised in Table 1. The 38 cyanobacterial strains could be assigned to 10 genera, including 6 potentially new genera with at least one species per genus. The 26 green algal strains fell into nine genera, including at least 10 species, out of which several species represent potentially new species. The 58 fungal isolates were distributed over 14 genera, including 17 species, of which one species is new to science.

**TABLE 1:** Overview of isolates and their ecological function collated from AlgaeBase (Guiry and Guiry 2025) for cyanobacteria and green algae and from the FungalTraits database (Põlme et al. 2020) for fungi.

Order	Genus	Species	# of isolates	Frequent habitat	General ecological function/lifestyle	Location			
						C (humid)	PB	LCI	KC (dry)
Filobasidiales	*Naganishia*	*albida*	1	Cosmopolitan	Pathogen		1		
Russulales	*Heterobasidion*		1	Cosmopolitan, terrestrial	Phytopathogen, decomposition		1		
Polyporales	*Phlebia*		1	Cosmopolitan, terrestrial	Phytopathogen, decomposition	1			
Pleosporales	*Preussia*	*australis*	14	Dry rainforest, Australia	Endophyte	5	4	3	2
Pleosporales	*Phytomyces*	*chartarum*	1	Subtropical to cosmopolitan	Phytopathogen, decomposition				1
Pleosporales	*Paraphaeosphaeria*		2	Europe/North America	Endophyte	1			1
Pleosporales	*Epicoccum*	*nigrum*	1	Cosmopolitan, soil	Endophyte, phytopathogen, decomposition			1	
Pleosporales	*Alternaria*		16	Cosmopolitan, soil	Saprophyte, phytopathogen, decomposition	5	6	3	2
Lecanicillium	*Lecanicillium*		1	Cosmopolitan	Ethnomopathogen	1			
Helotiales	*Botrytis*	*cinerea*	1	Cosmopolitan, terrestrial	Phytopathogen, decomposition				1
Eurotiales	*Penicillium*	*citreonigrum*	4	Cosmopolitan, soil	Saprotroph, endophyte			1	3
Eurotiales	*Penicillium*	*chrysogenum*	1	Cosmopolitan	Unknown			1	
Eurotiales	*Penicillium*	*brevicompactum*	1	Cosmopolitan, terrestrial	Unknown				1
Dothideales	*Aureobasidium*	*pollulans*	1	Cosmopolitan, terrestrial	Phytopathogen, endophyte				1
Capnodiales	*Constantinomyces*		1	Terrestrial, lithic	Saprotroph		1		
Capnodiales	*Cladosporium*	I	2	Terrestrial, soil	Saprotroph, phytopathogen, decomposition	2			
Capnodiales	*Cladosporium*	II	9	Terrestrial, soil	Saprotroph, phytopathogen, decomposition	2	4	1	2
Total isolates per site						17	17	10	14
Total fungal isolates						58			
Nostocales	*Nostoc*	I	1	Cosmopolitan	Carbon fixation, nitrogen fixation				1
Nostocales	*Nostoc*	II	1	Cosmopolitan	Carbon fixation, nitrogen fixation				1
Chroococcidiopsidales		I	2	This study	Carbon fixation				2
		II	1	This study	Carbon fixation			1	
		III	9	This study	Carbon fixation				9
		IV	1	This study	Carbon fixation	1			
		V	14	This study	Carbon fixation			3	11
Coleofasciculales	*Kastovskya*	*adunca*	6	Atacama Desert, soil, biocrust	Carbon fixation, erosion prevention				6
Chroococcales			2	Cosmopolitan	Carbon fixation	2			
Leptolyngbyales	*Myxacorys*	*chilensis*	1	Atacama Desert, soil, biocrust	Carbon fixation, erosion prevention				1
Total isolates per site						3	0	4	31
Total cyanobacterial isolates						38			
Prasiolales	*Pseudochlorella*		6	Temperate, polar, epilithic, soil	Carbon fixation	4	2		
Prasiolales	*Diplosphaera*		1	Cosmopolitan	Carbon fixation	1			
Prasiolales	*Pseudostichococcus*		3	Cosmopolitan	Carbon fixation			3	
Chlorocystidales	*Desmochloris*		1	Temperate, freshwater	Carbon fixation	1			
	*Klebsormidium*		2	Cosmopolitan, soil, biocrusts	Carbon fixation, erosion prevention	2			
Trebouxiales	*Lobosphaera*		2	Temperate, soil	Carbon fixation		1	1	
	*Pleurastrosarcina*		8	South America, soil	Carbon fixation		5		3
Chlorococcales	*Coccomyxa*		2	Cosmopolitan	Carbon fixation				2
Trebouxiales	*Myrmecia*		1	Cosmopolitan, soil, terrestrial	Carbon fixation	1			
Total isolates per Site						9	8	4	5
Total green algal isolates						26			
		Total Isolates	122						

In the following, the single groups are briefly presented with indications given about potentially novel clades, genera or species.

### 3.3 Cyanobacteria

**FIGURE 2:**
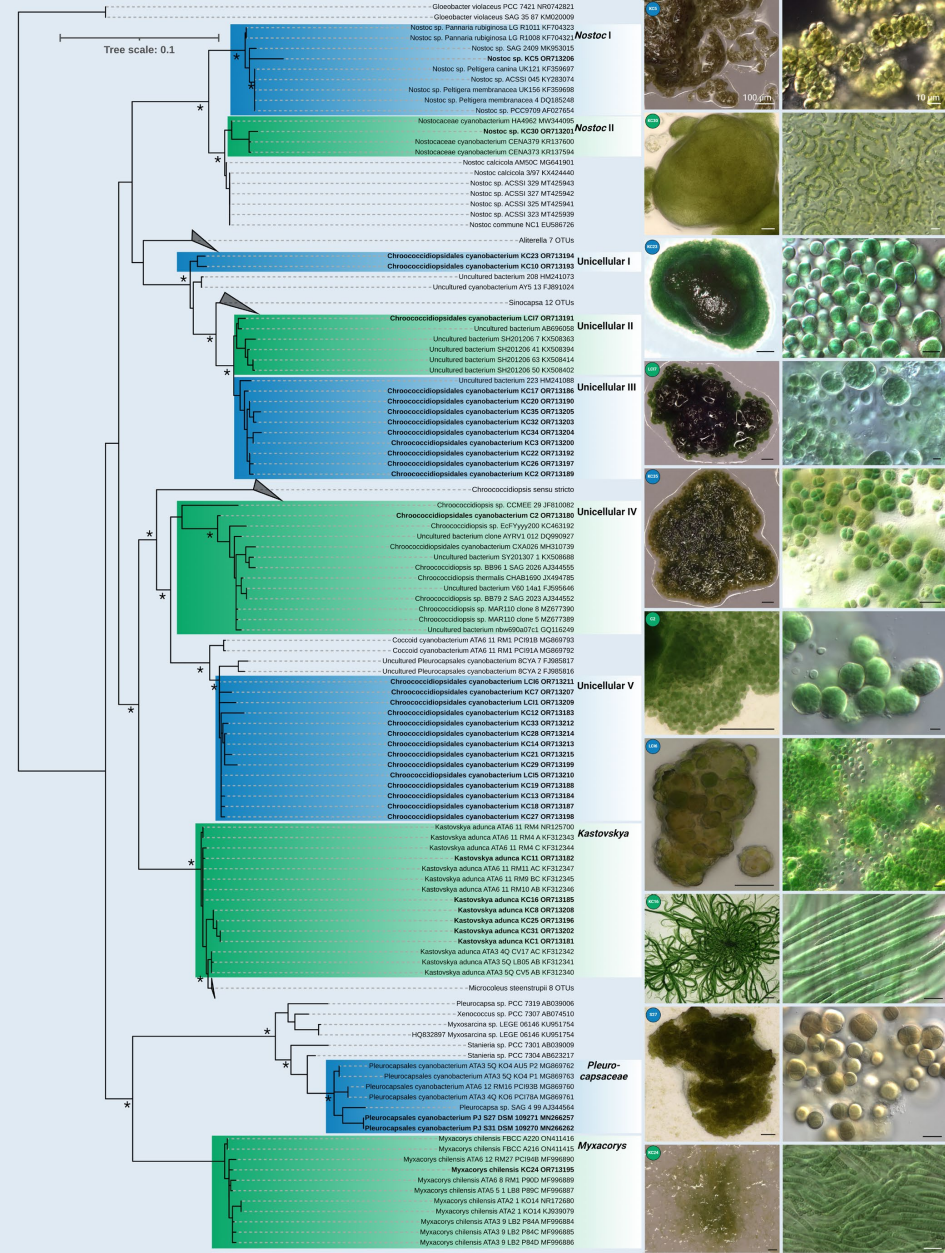
Molecular phylogeny of isolated cyanobacteria based on 16S rRNA gene region sequence analysis. A phylogenetic tree (left) was inferred by the Bayesian method with Bayesian posterior probabilities (PPs) and maximum likelihood bootstrap support (BP); branches supported in both analyses > 80% are labelled by an asterisk. Strains in bold represent newly sequenced algae. The right columns show micrographs of the isolated cyanobacteria with stereoscopic images taken directly from agar plates, followed by microscopy images.

Unicellular cyanobacteria from the order Chroococcidiopsidales were isolated, and the phylogenetic analysis positions them within five separate clades (Figure 2; unicellular I–V). This is in accordance with the morphology of all five clades, which resembled those of other chroococcidiopsidalean taxa (Figure 2). Significant differences in morphology between the five clades were detected by light microscopy, which distinguished the five clades based on variation in cell size, the formation of daughter cells and/or developmental stages (Figure 2). However, none of these five clades includes members of previously described genera or species such as *Aliterella*, *Gloeocapsopsis*, *Sinocapsa* or *Chroococcidiopsis sensu stricto*. The combination of the 16S rRNA gene region‐based position within the phylogenetic tree and the marked morphology indicated these five clades as potentially new genera.

Similarly, two unicellular isolates of the family Pleurocapsaceae within the order Chroococcales, PJS27 and PJS31, joined a well‐supported clade together with other 16S rRNA sequences. Those were generated from strains from other locations within the Atacama Desert (ATA‐strains) and one strain from hypolithic biofilms from stones of the Namib Desert (SAG 4.99), identifying this clade as a potential new genus of lithic desert environments.

In addition, filamentous, non‐heterocytous strains identified as *Kastovskya adunca* and *Myxacorys chilensis* were found (Figure 2).

Additionally, two heterocytous strains of *Nostoc* spp. were isolated, which joined separate clades and also differed in morphology by the formation of uniseriate vs. multiseriate filaments (Figure 2).

### 3.4 Green Algae

**FIGURE 3:**
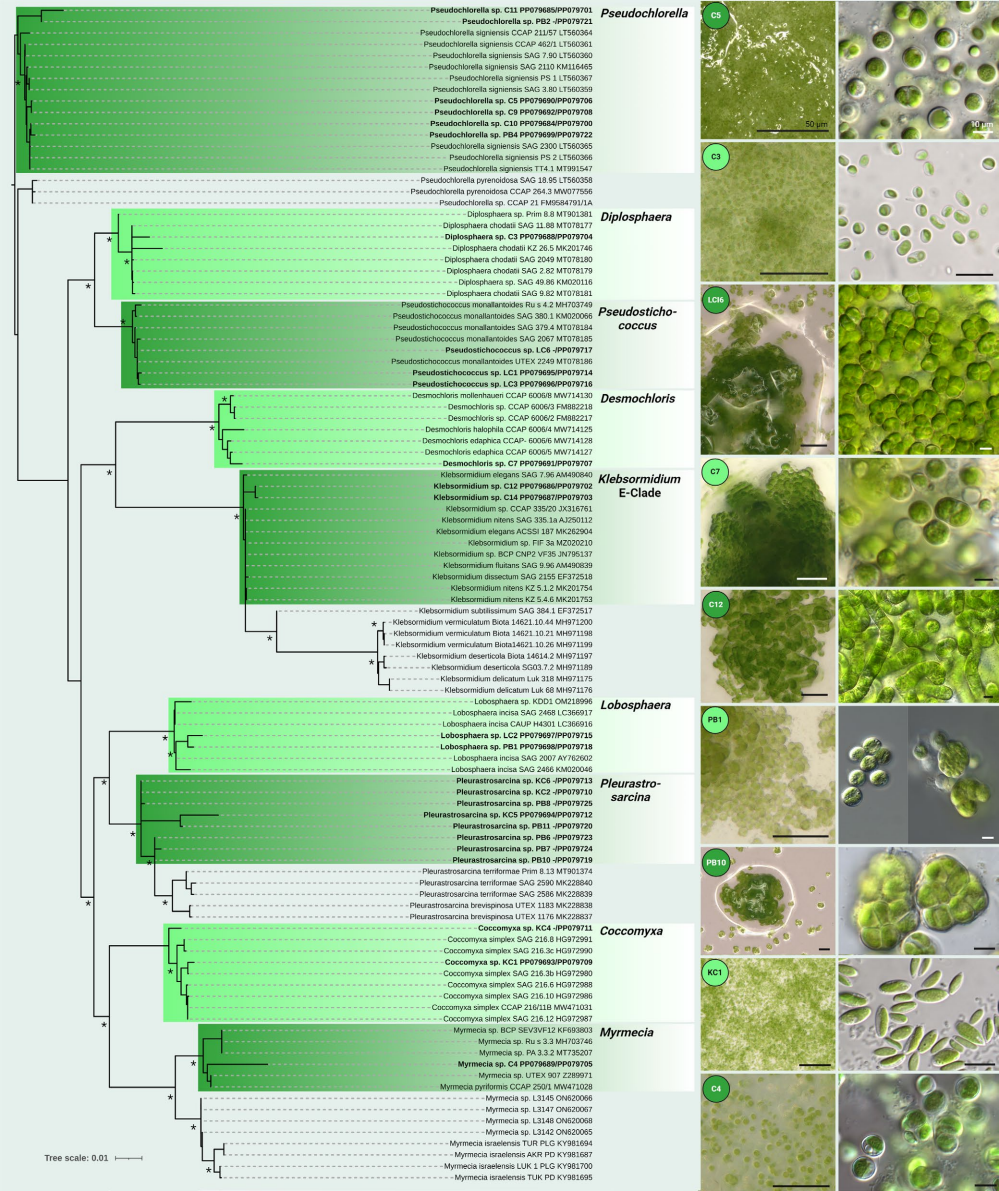
Molecular phylogeny of isolated green algae based on the concatenated 18S and SSU gene region sequence analysis. A phylogenetic tree (left) was inferred by the Bayesian method with Bayesian posterior probabilities (PPs) and maximum likelihood bootstrap support (BP); branches supported in both analyses > 80% are labelled by an asterisk. Strains in bold represent newly sequenced algae with their 18S rRNA sequence accession number, followed by the SSU sequence accession number. The right columns show micrographs of the isolated green algae with stereoscopic images taken directly from agar plates, followed by microscopy images.

Two filamentous green algae strains of the genus *Klebsormidium* were isolated, which fell in the E‐clade of the genus together with other members of the clade such as *K. elegans* , *K. nitens*, *K. fluitans* and *K. dissectum* (Figure 3). Based on the concatenated SSU and 18S rRNA gene region sequences, the strains were identical but differed in morphology as observed by microscopy. Strain C12, for example, showed more bloated cells, which were also wider than long, while strain C14 developed filaments with straight cell walls and isodiametric cells (Figure 3).

Several strains from the *Pseudochlorella signiensis* clade were isolated (Figure 3), which did not morphologically fit the description of *P. signiensis* sensu Darienko et al. (2016). Within this group, the two strains C11 and PB2 formed a sub‐clade compared to all other isolates. All strains investigated in this study differed from the description of the species *P. signiensis* by the commonly produced mucilage, the predominant formation of so‐called giant cells, and the frequent production of cell packages (Figure 3), all of which may indicate two potential new species.

Three strains were identified as *Pseudostichococcus* falling within the *P. monallantoides* clade (Figure 3). While *P. monallantoides* is described as forming longitudinal cells, our isolates exclusively formed round to ellipsoidal cells, including dense cell packages (Figure 3). Zoospores were not detected. However, species assignments within this genus are only possible based on a variety of culture conditions since the morphological plasticity of this genus is high (Proeschold and Darienko 2020).

Eight isolated green algal strains were identified as members of the recently emended genus *Pleurastrosarcina*, a unicellular alga occurring in arid deserts (Darienko et al. 2019). The eight isolates cluster with the genera *P. terriformae* and *P. brevispinosa*, and the morphology was similar to *P. terriformae var. sanctae‐gracia* (Darienko et al. 2019; Figure 3).

In addition, several other unicellular green algae were detected, which could be assigned to certain genera, all of which have lichenised members. Based on their position in the phylogenetic tree and morphology, these isolates were classified as members of the genera *Desmochloris*, *Lobosphaera*, *Coccomyxa*, *Diplosphaera* or *Myrmecia* (Figure 3). The latter formed a separate clade together with *M. pyriformis* CCAP 250/1 and several undescribed *Myrmecia* species, which were distantly related to *M. israelensis*. However, the morphological features described for *M. pyriformis* , such as oblongated cells or the formation of large autosporangia comprising a large amount of small autospores, were not detected.

### 3.5 Non‐Lichenised Fungi

**FIGURE 4:**
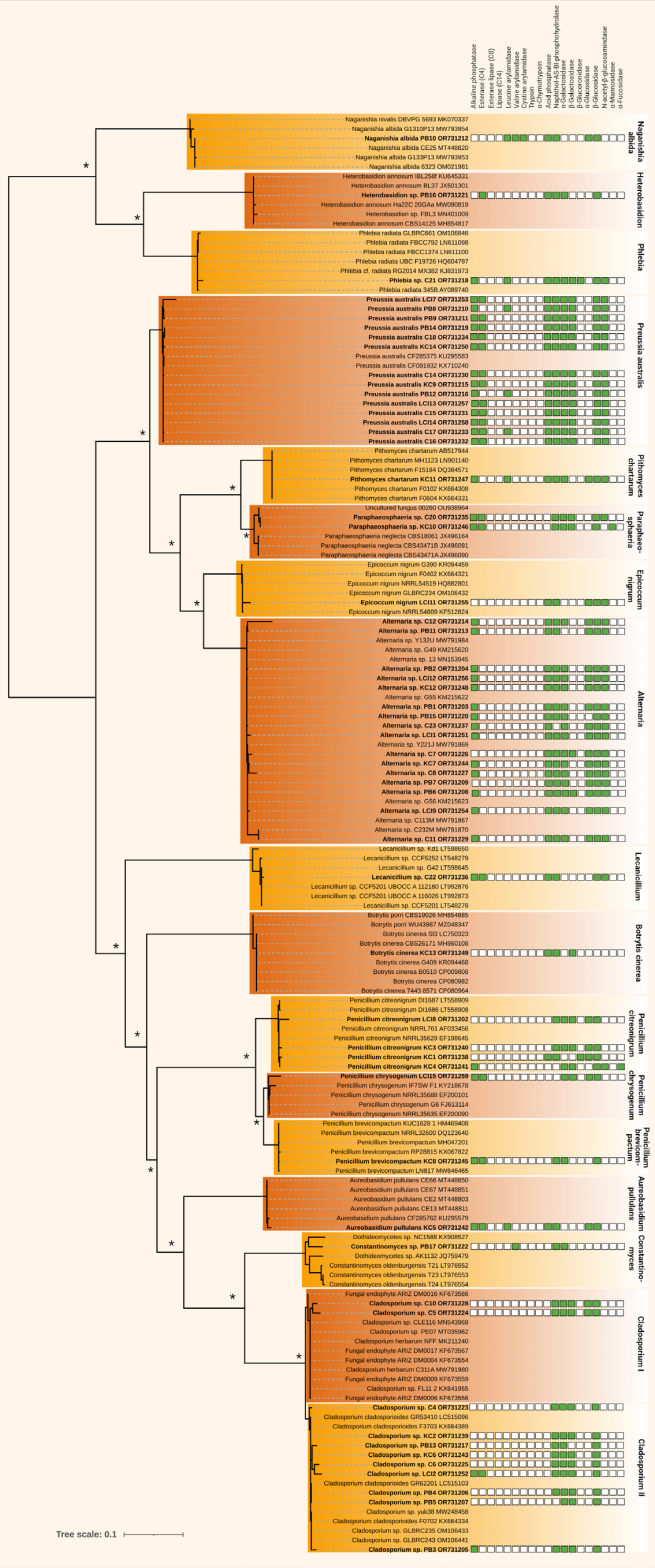
Molecular phylogeny of isolated free‐living fungi based on nuITS gene region sequence analysis and their enzyme activity. A phylogenetic tree was inferred by the Bayesian method with Bayesian posterior probabilities (PPs) and maximum likelihood bootstrap support (BP); branches supported in both analyses > 80% are labelled by an asterisk. Strains in bold represent newly sequenced fungi. Green boxes indicate activity of the corresponding enzyme.

Among the isolated non‐lichenised fungi, three isolates assigned to the genera *Phlebia*, *Heterobasidion* and *Naganishia* belonged to the basidiomycetes, while all the other 55 isolates represented ascomycetes (Figure 4).

The strain PB10 was assigned to the genus *N. albida* , a yeast‐like fungus forming cream to pale pink colonies with a smooth mucoid appearance.

The strain PB16 was characterised as a member of the *Heterobasidion annosum sensu lato* clade, which can be a saprotrophic and/or necrotrophic plant pathogen on dead wood or living roots.

This is comparable to strain C21, which was identified as a member of the genus *Phlebia* (Figure 4), a xylophagus crust fungus causing white rot on wood.

Most other isolates were assigned to *Preussia australis*, *Alternaria* spp. and two separate *Cladosporium* clades, *C. herbarum* (Cladosporium I) and *C. cladosporoides* (Cladosporium II), respectively. Together with other isolates identified as members of the genera and species *Paraphaeosphaeria*, *Epicoccum nigrum*, *Botrytis cinerea* and *Phytomyces chartarum*, they represented mostly endophytic plant pathogens.

We also isolated a strain which could be assigned to the genus *Constantinomyces* (Figure 4), a genus of melanised, slow‐growing and stress‐tolerant microcolonial ascomycetes. Only a few species within this genus have been described (Ruibal et al. 2018), but none from the Atacama Desert, indicating a potentially new species.

**FIGURE 5:**
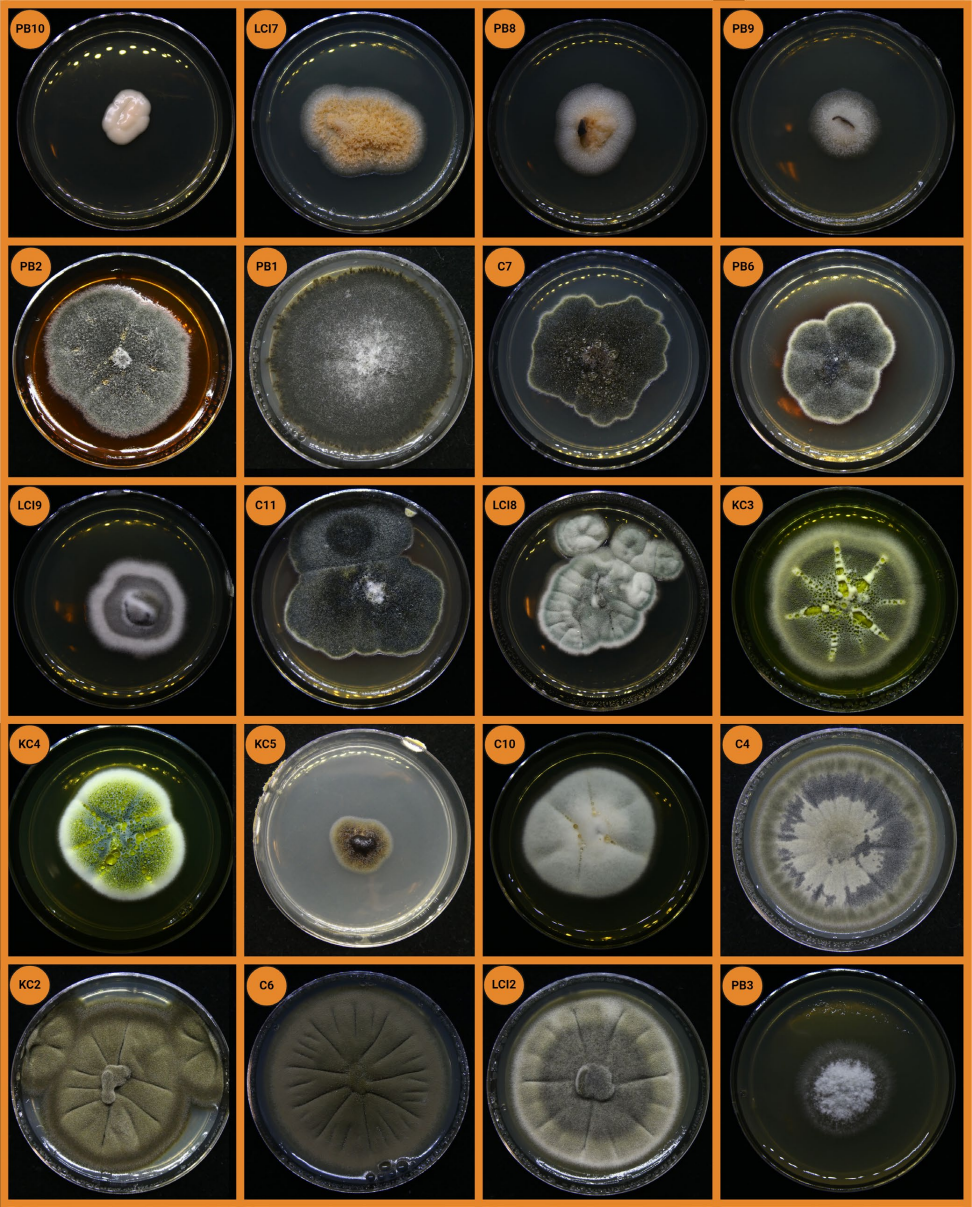
Photographs of isolated fungi. Isolates growing on Sabouraud agar plates with 4 cm diameter. Photos were taken after 5 days of growth at 25°C in the dark.

In addition, three different species of *Penicillium* were identified based on their phylogenetic position (Figure 4) and morphology (Figure 5), *P. chrysogenum*, *P. brevicompactum* and *P. citreonigrum*, all of which are known as plant pathogens.

All isolated fungi showed enzymatic activity, and most of them developed taxon‐specific activities (Figure 4). Most fungal isolates showed activity of the following enzymes: (i) alkaline phosphatase (hydrolysis of phosphate groups from different molecules under alkaline pH conditions; nutrient acquisition), (ii) esterase (C4) (hydrolysis of ester bonds present in lipids; symbiontic and pathogenic interactions), (iii) acid phosphatase (catalysation of hydrolysis of phosphate groups from different molecules under acid pH conditions; nutrient acquisition), (iv) naphtol‐AS‐BI‐phosphohydrolase (hydrolysis of phosphoric acid esters under alkaline conditions; nutrient acquisition, symbiontic and pathogenic interactions), (v) α‐galactosidase (hydrolysis of α‐galactosidic linkages in various carbohydrates; symbiontic and pathogenic interactions), (vi) β‐galactosidase (hydrolysis of β‐galactosidic linkages in various carbohydrates; symbiontic and pathogenic interactions), (vii) β‐glucosidase (hydrolysis of β‐glucosidic linkages in various carbohydrates; cellulose degradation, plant–fungal interactions, nutrient acquisition) and (viii) N‐acetyl‐β‐glucosaminidase (hydrolysis of N‐acetylglucosamine [GlcNAc] residues from amino sugars and complex carbohydrates; chitin degradation, symbiotic and pathogenic interactions).

In contrast, the saprophytic *Naganishia albida* PB10 was the only strain that tested positive for cystin arylamidase (hydrolysis of cystine or cysteine‐aryl amides; sulphur/nitrogen metabolism), which occurs rarely in fungi.

No isolated fungus tested positive for α‐chymotrypsin (degradation of extracellular proteins from organic matter; pathogenic interactions), which is in accordance with the usual absence of this enzyme in fungi.

## 4 Discussion

In the present study, we show a comprehensive analysis of the biodiversity of non‐lichenised cyanobacteria, green algae and fungi as an integral part of the grit crust microbiome from the coastal Range of the Atacama Desert spanning over four contrasting sites within the National Park Pan de Azúcar. This diversity has been captured using an integrative culture‐dependent approach, which considers morphological and molecular traits as well as ecosystem functions of the identified organisms. It should be noted that an isolation‐based workflow can underestimate microbial diversity in natural communities or can potentially lead to the overestimation of certain taxa, which can grow quickly in culture. In addition, underestimation or even failing to detect unculturable species, which could be dominant elements in natural biocrusts, is a common bias (Büdel et al. 2016; Samolov et al. 2020). However, our aim was to obtain live isolates for detailed morphological and phylogenetic analyses, which cannot be achieved through amplicon‐based molecular approaches alone. The culture‐based method allows for downstream experimental manipulation and reference genome sequencing, which is crucial for characterising novel taxa.

### 4.1 Non‐Lichenised Microalgal and Fungal Diversity

In contrast to all other biocrusts worldwide, the grit crust is established on and within 2–6 mm sized granitoid pebbles locally called *maicillo* (Jung, Mikhailyuk, et al. 2020). Comparable to hypolithic niches known globally from desert environments (Warren‐Rhodes et al. 2006; Chan et al. 2012), these translucent pebbles probably increased the possibility of fog and dew‐water condensation because of higher thermal conductivity, while also shielding the organisms from damaging solar radiation (Azúa‐Bustos et al. 2011), resulting in a unique microbiome compared to other biocrusts. This uniqueness was recently highlighted by investigations on the grit crust‐forming lichens with green algal photobionts, which are the predominant key organisms of this microbiome besides the non‐lichenised microorganisms (Jung, Brand, et al. 2024). This study demonstrated that the lichens exclusively partner with photobionts of the green algal genus *Trebouxia*, resulting in exceptionally high Chlorophyl_a+b_ values (420 mg Chl_a+b_ per m^2^), which reflects the high productivity of this soil ecosystem (Jung, Brand, et al. 2024). Additionally, the initial metabarcoding approach showed that 80% of the amplicon sequence variants (ASVs) corresponded to the photobionts, while the remaining ASVs were assigned to *Diplsophaera* spp., the *Stichococcus*‐complex, *Myrmecia*, *Elliptochloris* and several taxa reflecting less than 1% of the ASVs. Interestingly, our culture‐dependent approach recovered isolates from the main genera but also almost all genera falling within the less than 1% taxa. Among them were *Klebsormidium*, *Pseudochlorella*, *Desmochloris*, *Lobosphaera*, *Pleurastrosarcina* and *Coccomyxa* (Figure 3). This is remarkable because there is usually a large gap between amplicon sequencing‐derived biodiversity and culture‐dependent methodologies (Rippin et al. 2018), and it shows that rare taxa can also be isolated from such complex microbial systems.

In the previous study (Jung, Brand, et al. 2024), it was also reported that, besides *Trebouxia*, all other detected algal taxa were free‐living, since only members of the genus *Trebouxia* were found to be lichenised. Most green algal taxa isolated here have been found lichenised but also free‐living in other habitats (Fontaine et al. 2013), where they are probably growing epiphytically on the lichens or attached to the surface of the grit stones. This is of interest, for example, for the isolate *Myrmecia* sp. C4, which probably represents a new species. It is free‐living, a significant member of the grit crust microbiome and has a distinct morphology compared to other species. This isolate can help future studies resolve the perplexing taxonomy of this important but little‐studied facultatively lichenised algal genus, which is a frequent member of biocrusts (Moya et al. 2018; Samolov et al. 2020). This is comparable to the isolated *Pseudochlorella* strains, which corresponded to the species cluster of *P. signiensis*, which is also terrestrial/epilithic (Darienko et al. 2016), but the strains isolated from the grit crust show a different morphology (Figure 3). Interestingly, we also isolated members of the recently revised genus *Pleurastrosarcina*, where the species *P. terriformae var. sanctae‐gracia* was described from a biocrust part of a nature reserve several hundred kilometres further south of Pan de Azúcar (Darienko et al. 2019). Compared to the Atacama Desert, the nature reserve Sancta Gracia is semi‐arid, affected by rainfall reaching 83.4 mm year^−1^ and shows herbaceous vegetation (Samolov et al. 2020), which indicates that we may have isolated an additional variety of *P. terriformae* or a new species of the genus.

Of the 38 isolated cyanobacterial strains, most fell into the order Chroococcidiopsidales, which is a unicellular order comprised of the genera *Chroococcidiopsis*, *Gloeocapsopsis*, *Aliterella*, *Sinocapsa*, *Gloeocapsa* and others (summarised in Jung et al. 2021). None of the strains isolated during this study belongs to any of the described genera, which is interesting because, for example, *Aliterella chasmolithica* has been described from cracks in large rocks of the same habitat (Jung, Mikhailyuk, et al. 2020), and *Chroococcidiopsis* is also considered to be a cyanobacterial genus frequently found in deserts worldwide (Bahl et al. 2011). The five new genera, which could potentially be described in the future based on the set of isolates generated here, showed the distinctive niche the grit crust provides for these unicellular cyanobacteria. This is important because Chroococcidiopsidales have been shown to belong to the most extremotolerant members of the phylum (Azua‐Bustos et al. 2014; Li et al. 2022), designating them as an interesting objective for aspects of astrobiology (Billi 2019; Jung et al. 2022). In addition to the unicellular Chroococcidiopsidales, two strains of unicellular Chroococcales, family Pleurocapsaceae, were isolated, which fell into a separate clade together with others from the Atacama Desert and the strain 4.99 from the Namib Desert (Figure 2). The latter was isolated from hypolithic biofilms of a quartz pebble from Henties Baai (Büdel 1991). It has already been assumed that there is a great ecological overlap between the Namib and the Atacama Desert (Jung, Mikhailyuk, et al. 2020), and systems comparable to the grit crust have recently been found in the Namib Desert (De los Rios et al. 2022), but no significant organismic overlap has so far been detected. Further studies will show if the clade can be established as a novel genus supporting this congruence. Filamentous strains such as *M. chilensis* and *K. adunca* were also isolated from the grit crust, both previously recorded from the Atacama Desert and thought to be endemic (Pietrasiak et al. 2019; Mühlsteinová et al. 2014).

In general, the role of non‐lichenised fungi in biocrusts is not well studied; however, it has been suggested that fungal mycelia link biocrusts with patches of arid land vegetation, mediating nutrient exchange between these systems (Collins et al. 2008; Green et al. 2008). The initial metabarcoding conducted in Jung, Brand, et al. (2024) showed that the lichen mycobionts mainly accounted for the fungal ASV reads, but non‐lichenised members were also detected, such as Dothideomycetes and about 25% of unidentified fungi. The culture‐dependent approach applied here yielded, for example, a potential new species of Dothideomycetes within the genus *Constantinomyces* (Figures 4 and 5), a black microcolonial meristematic fungus. However, it should be mentioned that the chosen medium for the fungal enrichment cultures might have biased the isolated consortium towards fast‐growing strains, as a greater set of media allows for a more complete overview, including slow‐growing, microcolonial fungi (Muggia, Candotto‐Carniel, et al. 2017; Muggia, Kopun, et al. 2017).

### 4.2 Differences Between Sites

**FIGURE 6:**
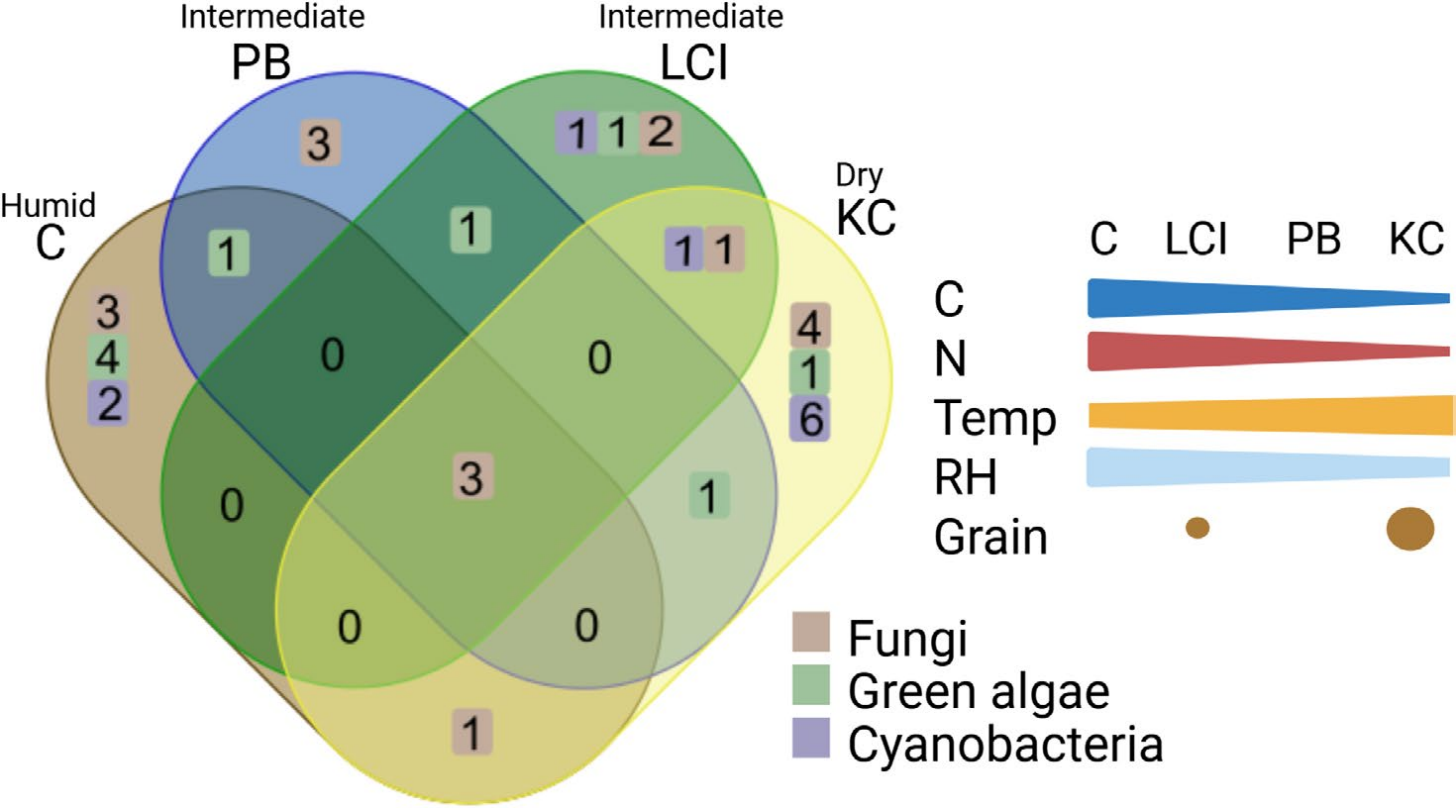
Venn diagrams showing the number of isolated cyanobacteria, green algae and fungi per site with a simplified scheme of the abiotic factors. C = carbon content; Grain = texture of the soil; N = nitrogen content; RH = mean relative air humidity; Temp = mean daily temperature.

The four sites were selected because they followed a water availability gradient ranging from site C (humid), close to the coast, where relative humidity rarely dropped below 36% to site KC (dry), further inland, representing the warmest and driest site. This trend was reflected in species composition as already presumed in Jung, Brand, et al. (2024), where site C was dominated by green algae leading to a high productivity shown by the highest chlorophyll amounts (Jung, Brand, et al. 2024), and as a result, also the highest carbon and nitrogen contents (Figures 1E and 6). At site C (humid), green algae and lichens dominate over cyanobacteria, while the latter take over at the driest site KC (Figure 6). The KC site is 12 km off the coast and is protected by surrounding hillslopes so that fog rarely reaches the area. Here, the relative air humidity ranging from 25% to 89% at ground level indicates that dense fog rarely reaches the site. These humidity levels only allow the establishment of a sparse lichen and green algal community, as was detected by metabarcoding (Jung, Brand, et al. 2024), while cyanobacteria are more dominant.

This might be due to water condensation on the grits, which allows the establishment of cyanobacterial‐dominated hypolithic biofilms (Azúa‐Bustos et al. 2011). Condensation of water is difficult to measure in nature (Lakatos et al. 2012). However, the humidity and temperature measurements indicate regular evening condensationand morning evaporation at all four sites on about half of the recorded days (Figure 1F). Nighttime cooling and post‐sunrise warming were generally continuous, but occasional evening heating and morning cooling “peaks” suggest condensation–evaporation cycles. Condensation releases latent heat, warming the substrate, while subsequent evaporation absorbs heat, cooling it. This is very interesting for the driest site, KC, for two reasons. First, this condensation cannot be measured with the usual standardised microclimate stations at a height of 2 m, but only directly on the ground surface. Second, the low humidity at the KC site shows that water ingress via fog is unlikely, but despite the low RH, condensation and thus regular water ingress still occurs due to the temperature amplitudes between air and ground. However, presumably less water condenses due to the lower humidity and the associated lower volume loading of the air with water droplets. The different form of water availability also explains the different colonisation patterns of the study areas. While lichens and green algae are photosynthetically activated by both high humidity and water droplets—as in the case of C (humid), LCI and PB, where they are dominant—cyanobacteria require liquid water (Lange et al. 1993; Gauslaa 2014). Cyanobacteria therefore do not find the more humid sites C, LCI and PB to have such suitable ecological niches, hence the dominance of lichens and green algae; however, they can successfully dominate at the driest Site KC due to the utilisation of the low condensates.

Moreover, at KC, bioweathering processes mediated by microorganisms contributing to the grit crust (Jung, Mikhailyuk, et al. 2020) might also be delayed due to restricted activity periods with regard to water availability, which is reflected in a coarser substrate (Figure 1D).

### 4.3 Functional Ecology and Diversity of the Culturable Grit Crust Microbiome

In general, biocrusts from South America are considered to be the most understudied biocrusts in terms of microbial diversity (Büdel et al. 2016); however, this was recently tackled by several studies detecting biocrusts along a Chilean precipitation and vegetation gradient (Baumann et al. 2018; Samolov et al. 2020). Regarding green algae, these studies mostly found unicellular, densely packed Trebouxiophyceae and Chlorophyceae, which supports biocrust research from North America (Lewis and Flechtner 2002). Besides their role as primary producers (Table 1), these unicellular green algae often excrete EPS which provide desiccation protection in arid environments (Knowles and Castenholz 2008) but also allow them to stick to dust particles, promoting their dispersal (Tesson et al. 2016). Filamentous green algae such as *Klebsormidium*, which was also detected in the present study, can support the stabilisation of soil particles (Table 1) and are also frequently part of biocrusts (Borchhardt and Gründling‐Pfaff 2020; Glaser et al. 2022). In other studies, *Klebsormidium* has been reported to replace cyanobacteria‐dominated biocrusts (Glaser et al. 2018) due to higher water input, and we observed a similar trend: green algae were most abundant at site C (humid) which was close to the coast with high fog water depositions compared to the dry site KC, 12 km off the coast, where mainly cyanobacteria were isolated. Usually, green algae and green algal lichens (chlorolichens) are more abundant in cold deserts where fog and dew represent the main water sources, such as in the Atacama or Negev Desert (Jung, Brand, et al. 2024; Kidron 2019). In contrast, cyanobacteria and cyanobacterial lichens (cyanolichens) dominate hot deserts with occasional rain events (Büdel et al. 2018). Cyanobacteria are usually the pioneers in desert environments and can be divided into certain groups with characteristics resulting in corresponding functional traits (Xiao et al. 2023). Examples of these are the filamentous *K. adunca* and *M. chilensis*, which are biocrust‐forming taxa, while nostocalean taxa such as the isolated *Nostoc* spp. can fix atmospheric nitrogen, enriching the surrounding soil through leaching (Dojani et al. 2007; Wu et al. 2023). Unicellular taxa such as Chroococcidiopsidales have been shown to play active roles during nutrient acquisition by shifting the pH of their substrate and bio‐weathering (Büdel et al. 2004). They also produce copious amounts of EPS, including sunscreen pigments such as scytonemin, which can protect the whole microbial community (Dillon et al. 2002). In most desert biocrusts, the bundle‐forming *M. vaginatus* and *M. steenstruppii* are dominant taxa (Moreira et al. 2021), but in the grit crust, they were not detected based on the isolation approach. Instead, the morphologically and phylogenetically closely related *K. adunca* was found, a species considered to be endemic to the Atacama Desert environment (Mühlsteinová et al. 2014). The geographical restriction of this genus appeared to hold true since *K. adunca* had not been detected during metabarcoding studies of other biocrust sites, for example, from the Ural Mountains (Patova et al. 2023), Pamir Mountains (Khomutovska et al. 2020) or the Sahara Desert (Mehda et al. 2021). However, recently, new species of the genus were described from various habitats, some of which were outside of the Atacama Desert (Jusko et al. 2024).

Non‐lichenised biocrust‐associated soil fungi can also be assigned to certain functional roles such as biological controllers (e.g., parasites and phytopathogens), ecosystem regulators (involved in carbon, nitrogen and phosphorus cycles) and organic matter decomposers (Treseder and Lennon 2015; Frąc et al. 2018). Fungi especially play important roles in maintaining ecosystem quality and functional diversity. Due to their ability to produce a wide variety of extracellular enzymes, fungi control organic matter degradation, mineralisation and nutrient recycling, and therefore regulate the balance of carbon and nutrients (Žifčáková et al. 2016; Stege et al. 2010). The different enzymes produced by the fungi detected here contribute to various ecosystem functions: almost all isolates tested positive for alkaline and acid phosphatases, which are involved in the release of phosphorus into the environment and therefore play important roles in the phosphorus cycle (Srivastava et al. 2021). Beta‐glucosidase activity has been found to be sensitive to soil management and has been proposed as a soil quality indicator because it provides an early indication of changes in organic matter status and its turnover. Research has shown that β‐glucosidase is the most abundant and easily detected of the three enzymes involved in cellulose degradation in soil and is an essential enzyme in the carbon cycle. The products of β‐glucosidase hydrolysis are useful as energy sources for microorganisms and plants (Stege et al. 2010). When released by fungi and other soil microorganisms, N‐acetyl‐β‐d‐glucosaminidase plays a vital role in increasing nitrogen remineralisation rates in soil by breaking down chitin into amino sugars. N‐acetyl‐β‐d‐glucosaminidase activity has been found to significantly correlate with increased total nitrogen mineralisation, organic carbon production and ammonium fixation in local environments. High levels of chitin occur in soil compounds globally; therefore, the ability of N‐acetyl‐β‐d‐glucosaminidase to break it down suggests that the enzyme is a key player in the transitioning of concentrations of nitrogen, carbon and ammonium in soil and soil‐dependent ecosystems (Ekenler and Tabatabai 2004; Miller et al. 1998). Our results show the high degree of endophytic and phytopathogenic lifestyles of the isolated biocrust‐associated fungi, which is probably due to the fact that most of them were associated with the lichens. As part of the lichen microbiome (Fernández‐Mendoza et al. 2017), these fungi might be involved in the symbioses, use the lichen as a substrate with access to carbohydrates and water, or play parasitic roles. A high degree of within‐lichen continuum dynamics between lichen mycobionts, photobionts and lichenicolous fungi, for example, has already been shown for this habitat (Jung et al. 2022).

However, the culturable microbiome of the grit crust now offers the unique opportunity for a diverse set of future studies on the isolated organisms. To our knowledge, such a span of taxa has so far not been isolated from any biocrust environment.

## 5 Outlook/Synthesis

This study elucidated the culturable microbiome of a desert ecosystem with a focus on non‐lichenised cyanobacteria, green algae and fungi, resulting in a unique set of well‐characterised genera and species, but also new as yet undescribed ones. This culture collection will allow detailed examination of these strains and the subsequent description of new taxa with important functions in comparable biocrust ecosystems. Complementary to this study, a follow‐up could focus on culturable heterotrophic bacteria, as their diversity within the grit crust remains unexplored. The cultures can also be used to individually investigate adaptation strategies to the abiotic conditions present in the Atacama Desert, which can now be carried out based on genome datasets derived from selected isolates. The presented data are also a reference for future explorations of other grit crust habitats in Chile outside of the National Park Pan de Azúcar, or for comparable biomes such as the Namib Desert in South Africa.

## Author Contributions

P.J. and M.L. took samples, generated funding and supervised the work. P.J. wrote the manuscript and prepared the figures, while E.J., L.W., L.B.‐W., R.B. and K.B. conducted lab work. All authors contributed to and edited the manuscript.

## Conflicts of Interest

The authors declare no conflicts of interest.

## Data Availability

The data that support the findings of this study are openly available in NCBI at https://www.ncbi.nlm.nih.gov/.
